# Strontium Chloride Improves Reproductive Function and Alters Gut Microbiota in Male Rats

**DOI:** 10.3390/ijms241813922

**Published:** 2023-09-10

**Authors:** Xulai Huang, Yanan Gao, Yangdong Zhang, Jiaqi Wang, Nan Zheng

**Affiliations:** 1 Key Laboratory of Quality & Safety Control for Milk and Dairy Products, Ministry of Agriculture and Rural Affairs, Institute of Animal Sciences, Chinese Academy of Agricultural Sciences, Beijing 100193, China; 2 Laboratory of Quality and Safety Risk Assessment for Dairy Products, Ministry of Agriculture and Rural Affairs, Institute of Animal Sciences, Chinese Academy of Agricultural Sciences, Beijing 100193, China; 3 Milk and Milk Products Inspection Center, Ministry of Agriculture and Rural Affairs, Institute of Animal Sciences, Chinese Academy of Agricultural Sciences, Beijing 100193, China; 4 State Key Laboratory of Animal Nutrition, Institute of Animal Sciences, Chinese Academy of Agricultural Sciences, Beijing 100193, China

**Keywords:** strontium chloride, reproductive function, gut microbiota, sperm quality, testosterone

## Abstract

Strontium (Sr) is an essential trace element in the human body and plays an important role in regulating male reproductive health. Recent studies have shown that gut flora plays a key role in maintaining spermatogenesis, as well as testicular health, through the gut–testis axis. At present, it is unclear whether gut microbiota can mediate the effects of Sr on sperm quality, and what the underlying mechanisms may be. We investigated the effects of different concentrations of strontium chloride (SrCl_2_) solutions (0, 50, 100, and 200 mg/kg BW) on reproductive function and gut microbiota in male Wistar rats (6–8 weeks, 250 ± 20 g). All the animals were euthanized after 37 days of treatment. The Sr-50 group significantly increased sperm concentration, sperm motility, and sperm viability in rats. After Sr treatment, serum and testicular testosterone (T) and Sr levels increased in a dose-dependent manner with increasing Sr concentration. At the same time, we also found that testicular marker enzymes (ACP, LDH) and testosterone marker genes (*StAR*, *3β-HSD*, and *Cyp11a1*) increased significantly in varying degrees after Sr treatment, while serum NO levels decreased significantly in a dose-dependent manner. Further investigation of intestinal flora showed that SrCl_2_ affected the composition of gut microbiome, but did not affect the richness and diversity of gut microbiota. Sr treatment reduced the number of bacteria with negative effects on reproductive health, such as *Bacteroidetes*, *Tenericutes*, *Romboutsia*, *Ruminococcaceae_UCG_014*, *Weissella*, and *Eubacterium_coprostanoligenes_group*, and added bacteria with negative effects on reproductive health, such as *Jeotgalicoccus*. To further explore the Sr and the relationship between the gut microbiota, we conducted a Spearman correlation analysis, and the results showed that the gut microbiota was closely correlated with Sr content in serum and testicular tissue, sex hormone levels, and testicular marker enzymes. Additionally, gut microbiota can also regulate each other and jointly maintain the homeostasis of the body’s internal environment. However, we found no significant correlation between intestinal flora and sperm quality in this study, which may be related to the small sample size of our 16S rDNA sequencing. In conclusion, the Sr-50 group significantly increased T levels and sperm quality, and improved the levels of testicular marker enzymes and testosterone marker genes in the rats. Sr treatment altered the gut flora of the rats. However, further analysis of the effects of gut microbiota in mediating the effects of SrCl_2_ on male reproductive function is needed. This study may improve the current understanding of the interaction between Sr, reproductive health, and gut microbiota, providing evidence for the development of Sr-rich foods and the prevention of male fertility decline.

## 1. Introduction

Infertility is a global problem and the World Health Organization predicts that it will become the third most common disease in the 21st century after cancer and cardiovascular disease. Infertility affects approximately 8 to 12 percent of couples of reproductive age, with male factors contributing to 50 percent of the total [1]. The social and economic burden caused by male infertility is increasing [2]. A recent comprehensive meta-analysis found that sperm counts (including sperm concentration and total sperm count) of men decreased significantly from 1973 to 2011, and decreased by 50–60% when unselected for fertility [3]. In addition, cross-sectional studies have found that when young men are diagnosed with infertility, their health is not as good as that of fertile men of the same age, indicating that men’s reproductive health may be closely related to their physical health [4,5]. Therefore, preventive and therapeutic factors are becoming increasingly important. In addition to medication, nutritional intervention would be beneficial to maintain male reproductive health.

Sr is an alkaline earth metal whose content is second only to calcium (Ca) in the earth’s crust [6]. Its mass fraction in the human body is not less than 0.05%, which is of great significance to human health. Many studies have shown that Sr has antioxidant [7], anti-inflammatory [8], anti-cancer [9], and anti-diabetes [10] properties and promotes angiogenesis [11,12]. A recent study found that the concentration of Sr in urine was positively correlated with sperm count, concentration, and activity, indicating that non-radioactive Sr in the form of Sr^2+^ has a beneficial effect on sperm quality [13]. Additionally, studies in humans, pigs, and rats have found that Sr^2+^ was important for maintaining reproductive health. In humans, Sr can support events related to the activation of human sperm capacitation, including protein tyrosine phosphorylation, overactivation, and recognition binding to zona pellucida [14]. In a pig model, it was found that Sr^2+^ had a positive effect on the fertilization of porcine sperm [15]. In a rat model, Sr fructose 1,6-diphosphate (FDP-Sr) was found to not only alleviate adenine-induced gonadal function in men but also to increase epididymal sperm count and testicular enzyme activity, increase T content, and improve testicular function [16]. The study found that in addition to the amount of Sr in the diet, the amount of Sr in the human body was also affected by sex and age [17,18]. Insufficient Sr intake may cause metabolic disorders, limb weakness, and other symptoms, while excessive Sr intake may lead to hypocalcemia and rickets [19]. However, there is no clear standard on the limit of Sr. Therefore, the safe limit of Sr in food, the optimal intake, and the role and mechanism of action in human health need further study.

There is a complex dynamic balance between intestinal microorganisms and the host. The gut microbiota is the second largest genome of the host, which can not only interact with the local intestinal tract, but also affect the physiological functions of distal organs such as the liver [20], brain [21], muscle [22], kidney [23], and testicles [24], which is of great significance for maintaining host health [25]. The gut microbiota contributes to the regulation of reproductive hormone secretion and is a potential biomarker of male hypogonadism, which is very important in the maintenance of male fertility and spermatogenesis [26,27,28,29]. For example, in azoospermia models, alginate oligosaccharides can rescue spermatogenesis damage by increasing *Bacteroidales* and *Lactobacillaceae* and by decreasing *Desulfovibrionaceae* [30,31]. In a testicular injury model, *Lactobacillus plantarum TW1-1* was found to regulate the intestinal microbiota in mice and prevent testis injury caused by diethylhexylphthalate (DEHP) [32]. In a diet-induced obesity (DIO) model, it was found that *Lactobacillus rhamnosus PB01* could significantly improve sperm motility and sperm parameters in mice [33]. A recent study showed that gut microbiota can improve male fertility by producing amino acid metabolites or transferring certain organs [34]. In addition, the gut microbiota can also regulate the permeability of the blood–testis barrier (BTB), which is important in testicular endocrine function [35].

However, the effect of Sr exposure on the gut microbiota of male rats has not been reported, and whether Sr can improve sperm quality in normal rats by regulating gut microbiota remains unknown. Therefore, the purpose of this study was to investigate the effects of Sr on sperm quality, gut microbiota composition, and structure in normal adult rats to provide a scientific basis for the development of Sr-rich foods and the application of Sr in nutrition.

## 2. Results

### 2.1. Sr Exposure Increased Rat Sperm Quality

**Figure 1:**
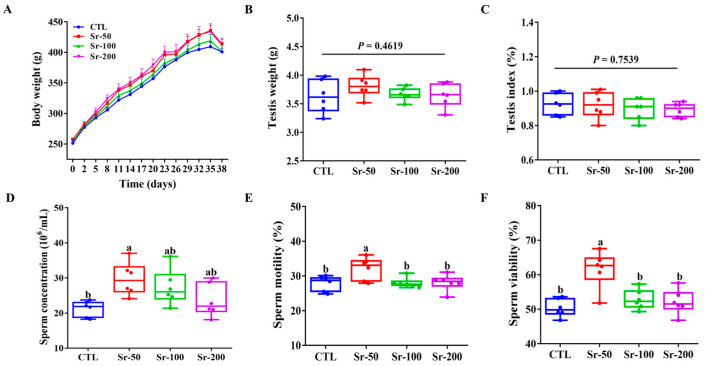
Effects of Sr exposure on the growth and sperm quality in rats: (**A**) Relative body weight changes during gavage in each group of rats. (**B**) Testis weight. (**C**) Testis index. (**D**–**F**) Sperm concentration, motility, and viability. The data are presented by mean ± SEM (*n* = 6). Significant differences between groups are indicated by different letters (a, b, c, d) (*p* < 0.05).

As shown in Figure 1A, the Sr-exposed group gained more weight over time compared to the control group. However, there were no significant differences in body weight, testicular weight (*p* = 0.4619), and testicular index (*p* = 0.7539) among the groups (Figure 1B,C). Compared with other groups, the Sr-50 group showed significantly increased sperm concentration, sperm motility, and sperm motility (*p* < 0.05), while the CTL, Sr-100, and Sr-200 groups had no significant effect on sperm concentration, sperm motility, and sperm viability compared to the control group (*p* > 0.05, Figure 1D–F). These results indicated that Sr exposure did not affect rat growth and could improve sperm quality.

### 2.2. Sr Exposure Increased the Levels of Marker Enzymes and Testosterone Synthesis Marker Genes and Decreased the Levels of Nitric Oxide Synthase (NOS) and Nitric Oxide (NO)

**Figure 2:**
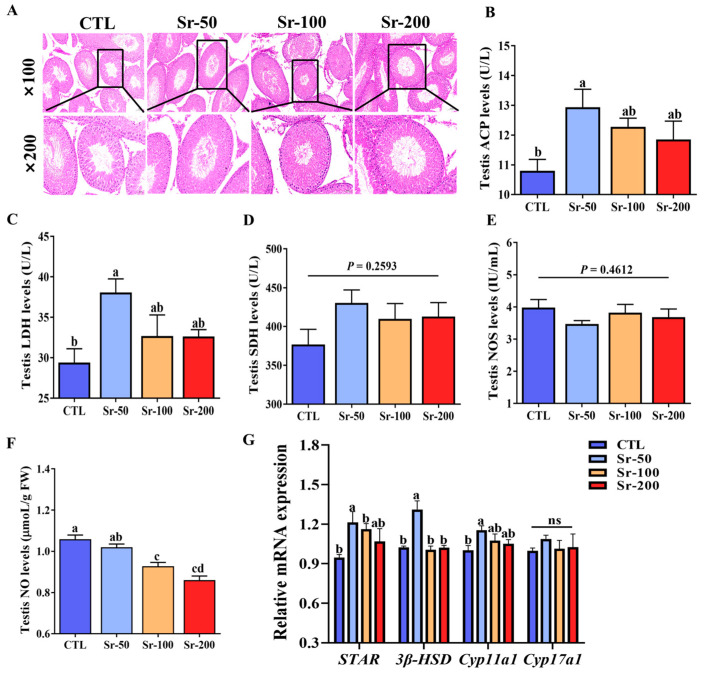
Effects of Sr exposure on testicular structure and function in rats: (**A**) Representative images of testicular tissue stained with H&E (100× and 200× magnifications). Bars represent 50 μm and 100 μm, respectively. (**B**) Testis ACP levels. (**C**) Testis LDH levels. (**D**) Testis SDH levels. (**E**) Testis NOS levels. (**F**) Testis NO levels. (**G**) Relative mRNA expression of *StAR*, *3β-HSD*, *Cyp11a1*, and *Cyp17a1* in the testis. Data are presented as mean ± SEM (*n* = 6). Significant differences between groups are indicated by different letters (a, b, c, d) (*p* < 0.05).

Histopathological examination results demonstrated that the testicular tissue structure of the control group and the Sr-exposed group was normal. The results showed that Sr exposure did not cause testicular damage (Figure 2A). Acid phosphatase (ACP), lactate dehydrogenase (LDH), and succinate dehydrogenase (SDH) are important enzymes involved in testicular metabolism. As shown in Figure 2B,C, the expression level of testis ACP and LDH increased significantly after Sr exposure (*p* < 0.05). According to Figure 2D, the expression of testis SDH levels in the CTL group was lower than that in the Sr-exposed groups, and the differences between the groups were not significant (*p* > 0.05). NOS and NO are important in reproductive activities such as androgen formation, sperm maturation, motility, and capacitation. However, we found no significant difference in testicular NOS expression levels between groups (*p* > 0.05, Figure 2E). Surprisingly, compared with the CTL group, testicular NO expression levels were significantly reduced in a dose-dependent manner (*p* < 0.05, Figure 2F). Genes such as steroidogenic acute regulatory (*STAR*), 3β-hydroxysteroid dehydrogenase (*3β-HSD*), P450 cholesterol side-chain cleavage (*Cyp11a1*), and cytochrome P450 17A1 (*Cyp17a1*) are important for the synthesis of T in the testis. Expression of *STAR*, *3β-HSD*, and *Cyp11a1* increased significantly (*p* < 0.05, Figure 2G) in the Sr-50 group. However, there was no significant difference in the expression of *Cyp17a1* among all groups (*p* > 0.05, Figure 2G). Thus, Sr improved sperm quality by increasing the levels of testicular marker enzymes and T synthesis marker genes and by decreasing the levels of NOS and NO.

### 2.3. Sr Exposure Increased Hormone Levels and Systemic Sr Content, but Its Significance Needs to Be Further Evaluated

**Figure 3:**
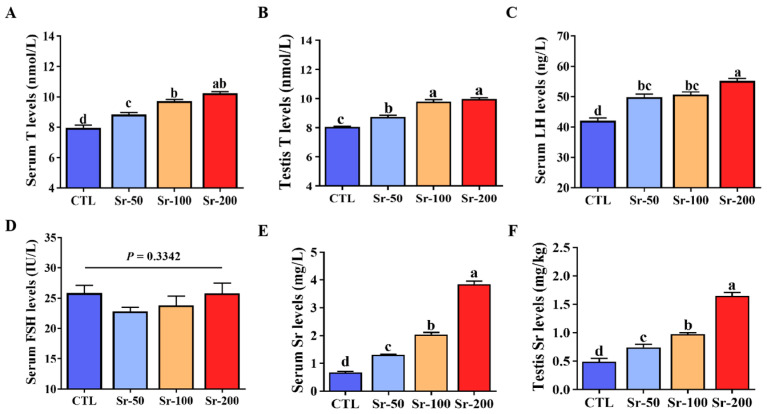
Effects of Sr exposure on hormone levels in rats: (**A**,**B**) Serum and testis T levels. (**C**,**D**) Serum LH and FSH levels. (**E**,**F**) Serum and testis Sr levels. Data are presented as mean ± SEM (*n* = 6). Significant differences between groups are indicated by different letters (a, b, c, d) (*p* < 0.05).

We found that Sr exposure significantly increased T (serum and testis) and luteinizing hormone (LH) levels in a dose-dependent manner compared with the CTL group (*p* < 0.05, Figure 3A–C). However, Sr exposure had no significant effect on follicle-stimulating hormone (FSH) levels in rats (*p* > 0.05, Figure 3D). In addition, we detected the presence of Sr in serum and testes. We found a significant increase in serum and testicular Sr content in the Sr-treatment groups compared to the CTL group in a dose-dependent manner (Figure 3E,F, *p* < 0.05). This suggests that Sr can enter the bloodstream through the intestinal tract, is transported to the testicular tissue, and accumulates in the testicular tissue.

### 2.4. Sr Exposure Does Not Affect the Richness and Diversity of Gut Microbiota

**Figure 4:**
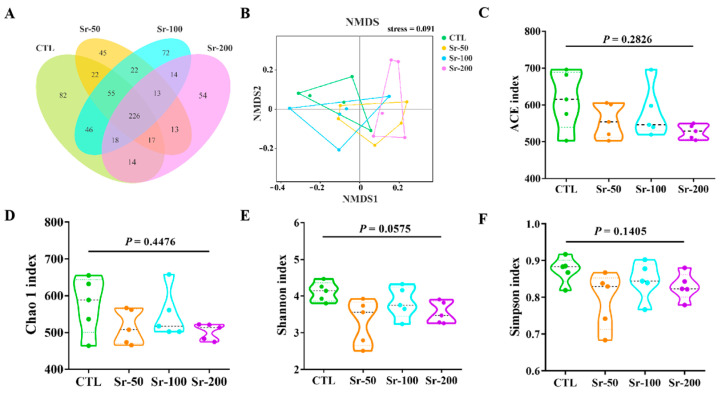
Effects of Sr exposure on the intestinal flora diversity of rats: (**A**) Venn diagram; (**B**) NMDS of the unweighted UniFrac distances; (**C**–**F**) ACE, Chao 1, Shannon, and Simpson indices.

To study the regulatory effect of Sr exposure on the normal host intestinal microbiome, cecal contents were collected and sequenced on an Illumina HiSeq platform to analyze the community structure. It was found that most of the OTUs (226 of 713) were shared by all groups, but some specific OTUs remained in the control group and the Sr exposure group (Figure 4A). According to the results of nonmetric multidimensional scaling (NMDS), the gut microbiota structures of the Sr exposure and CTL groups were significantly separated, indicating differences in the gut microbiota structure between the two groups (Figure 4B). We found that Sr exposure had no significant effect on the Alpha diversity index (including ACE, Chao1, Shannon, and Simpson) of normal rats, suggesting that Sr exposure did not affect the richness and diversity of intestinal microbiota (*p* > 0.05, Figure 4C–F).

### 2.5. Sr Exposure Specifically Altered Microbial Taxonomic Profiles

**Figure 5:**
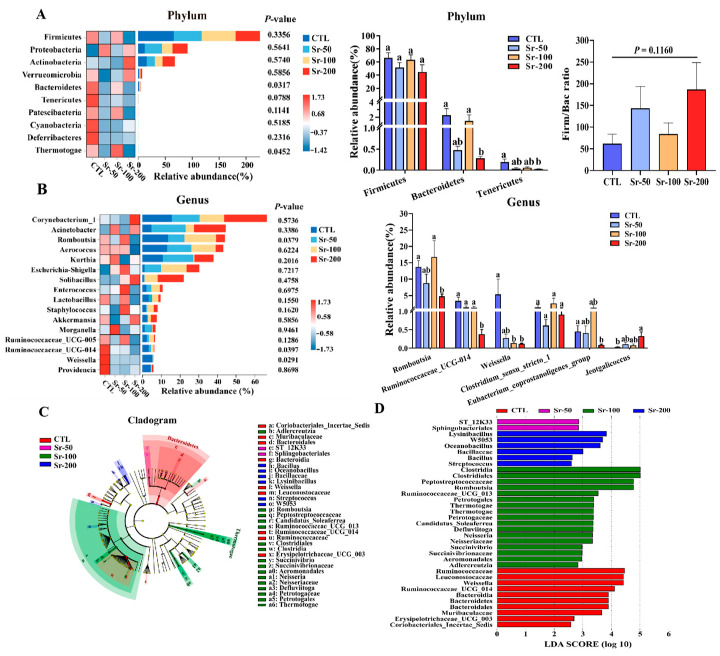
Effects of Sr exposure on the gut microbiota structure of rats: (**A**,**B**) Dynamic heat map stacks showing differences in the bacterial abundance between different at the phylum level (top 10) and genus level (top 16), and the relative amount of individual microbiota at the phylum level (top 10) and genus level (top 32). Relative abundance of phylum and genus microorganisms; (**C**) Cladogram of the CTL group vs. Sr-50 group vs. Sr-100 group vs. Sr-200 group; (**D**) Linear discriminate analysis (LDA) distribution of the CTL group vs. Sr-50 group vs. Sr-100 group vs. Sr-200 group. The LDA score threshold was 2.0. LDA effect size (LEfSe) was used to determine whether there were differences in microbial abundance. The data are presented as mean ± SEM (*n* = 5). Significant differences between groups are indicated by different letters (a, b, c, d) (*p* < 0.05). Wilcoxon rank sum test and Kruskal–Wallis rank sum tests were used for analysis.

To further research the effects of Sr exposure on intestinal microorganisms of normal hosts, the distribution of bacteria was discussed at the level of phylum and genus levels. In all groups, the dominant taxa in the rat gut microbiota came from three main phyla: *Firmicutes*, *Proteobacteria*, and *Actinobacteria* (Figure 5A). In the top 10 phyla, there were two bacteria with obvious differences in the relative abundance between the four groups, namely, *Bacteroidetes* (*p* = 0.0317) and *Thermotogae* (*p* = 0.0452) (Figure 5A). At the phylum level, *Firmicutes* of each group showed no significant change (*p* > 0.05). Sr treatment significantly reduced the expression of *Bacteroidetes* and *Tenericutes* (*p* < 0.05). Sr exposure increased the *Firm/Bac* ratio, but no significant difference was observed (*p* > 0.05).

In all samples, *Corynebacterium_1*, *Acinetobacter*, *Romboutsia*, *Aerococcus*, *Kurthia*, *Escherichia-Shigella*, and *Solibacillus* were the main dominant genera (Figure 5B). Among the top 16 genera, there were three species with significant differences in relative abundance among the four groups, namely *Romboutsia* (*p* = 0.0379), *Ruminococcaceae_UCG-014* (*p* = 0.0397), and *Weissella* (*p* = 0.0291). At the genus level (Figure 5B), Sr treatment significantly decreased the expression of the microorganisms such as *Romboutsia*, *Ruminococcaceae_UCG_014*, *Weissella*, and *Eubacterium_coprostanoligenes_group* (*p* < 0.05). The expression of bacteria such as *Jeotgalicoccus* was increased (*p* < 0.05).

The cladogram (Figure 5C) showed significant changes in gut microbiota at different taxa (phylum to genus) after Sr exposure, and the LDA score histogram (Figure 5D) presents differential bacteria in each group. It should be noted that among the 35 differential bacteria listed in Figure 5D, *ST_12K33*, and *Sphingobacteriales* were specific to the Sr-50 group. *Clostridia*, *Clostridiales*, *Peptostreptococcaceae*, *Romboutsia*, *Ruminococcaceae_UCG_013*, *Petrotogales*, *Thermotogae* (family and class), *Petrotogaceae*, *Candidatus_Soleaferrea*, *Defluviitoga*, *Neisseria*, *Neisseriaceae*, *Succinivibrio*, *Succinivibrionaceae*, *Aeromonadales,* and *Adlercreutzia* were specific to the Sr-100 group. *Lysinibacillus*, *W5053*, *Oceanobacillus*, *Bacillus*, and *Streptococcus* were specific to the Sr-200 group. The results showed that the dominant bacteria of the Sr-treated groups and the control group were different, and the dominant bacteria between the Sr-treated groups also changed with different doses. Therefore, these data indicate that SrCl_2_ treatment can modulate the composition of intestinal flora in normal rats.

### 2.6. Correlation between the Gut Microbiota and Host Phenotype

**Figure 6:**
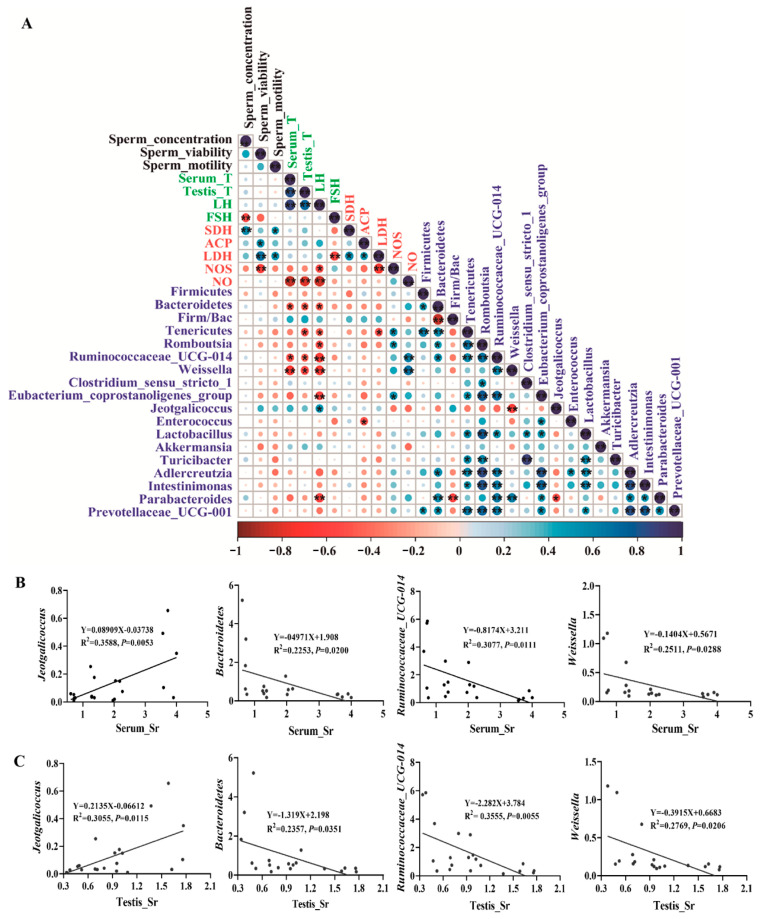
Correlation analysis of gut microbiota and host phenotype: (**A**) Spearman’s correlations between gut microbiota, sperm quality parameters, sex hormone levels, and testicular marker enzymes. The red and blue circles represent negative and positive correlations, respectively, while the large and small circles represent strong and weak correlations, respectively. The black font represents the parameters of sperm quality, the green font represents sex hormones, the red font represents testicular marker enzymes, and the blue font represents gut microbiota. * *p* < 0.05, ** *p* < 0.01. (**B**,**C**) The simple linear regression analysis was performed between representative microbe and Sr content in serum and testes (*n* = 5).

To further study the correlation between sperm quality parameters, hormone levels, testicular marker enzymes, and key gut microbial taxa, we conducted Spearman correlation analysis using the important indicators related to sperm quality of bacteria that showed significant changes at the species or genus level (Figure 6A, *p* < 0.05). *Jeotgalicoccus* was positively correlated with LH level (Figure 6A, *p* < 0.05). However, *Bacteroidetes*, *Tenericutes*, *Romboutsia*, *Ruminococcaceae_UCG_014*, *Weissella*, *Eubacterium_coprostanoligenes_group*, and *Parabacteroides* were negatively correlated with hormone levels (Figure 6A, *p* < 0.05). Additionally, we analyzed the relationship between the *Firm/Bac* ratio, sperm quality parameters, and testicular function due to its critical role in maintaining host health [36]. We found that sperm quality and testicular health were positively correlated with the *Firm/Bac* ratio (Figure 6A, *p >* 0.05). Moreover, we found that most of the bacteria have significant correlations with each other.

*Jeotgalicoccus* is the only beneficial bacteria found to be associated with a phenotype in this screening. On the other hand, *Bacteroidetes*, *Ruminococcaceae_UCG_014*, and *Weissella* are identified as the most harmful bacteria in phenotypic indexes. To gain deeper insights into the correlation between Sr and intestinal flora, we analyzed the Sr content of several bacteria and serum and testis in detail. There was a significant positive correlation between *Jeotgalicoccus* and Sr in serum (R^2^ = 0.3588, *p* = 0.0053) and testis (R^2^ = 0.3055, *p* = 0.0115) (Figure 6B,C, *p* < 0.05). Conversely, *Bacteroidetes* (R^2^ = 0.2253, *p* = 0.0200; R^2^ = 0.2357, *p* = 0.0351), *Ruminococcaceae_UCG_014* (R^2^ = 0.3077, *p* = 0.0111; R^2^ = 0.3555, *p* = 0.0055), and *Weissella* (R^2^ = 0.2511, *p* = 0.0288; R^2^ = 0.2769, *p* = 0.0206) exhibited a negative correlation with the Sr content in serum and testis (Figure 6B,C, *p* < 0.05). In summary, these results suggested that the crucial gut microbiota changes following Sr treatment played an important role in regulating male reproductive health. Further investigation of how these bacteria interact with the host is warranted.

## 3. Discussion

Sr has been shown to have a variety of biological functions, which are important for the maintenance of male reproductive health. An increasing number of studies have found that there is a close relationship between gut microbiota and reproductive health [37,38]. However, it is not known whether Sr can improve the sperm quality of normal hosts and whether its effects are related to the changes in intestinal flora. In this study, the effects of Sr treatment on the sperm quality and gut microbiota of the normal host were investigated.

Firstly, we found that Sr exposure did not affect the body weight, testicular weight, or testicular index in rats, indicating that Sr exposure did not negatively impact normal rat growth and development. Secondly, we found that Sr supplementation for 37 days in rats improved the sperm concentration, sperm motility, and sperm viability of rats, and the effect of the Sr-50 group was significant. It shows that Sr has great potential in improving male reproductive ability and is worth further exploration.

Diet is the main source of trace elements in the animal body. Previous studies have reported that there is a linear positive correlation between the intake of dietary trace elements and their serum levels [39,40]. The results of the present study found a positive correlation between Sr levels in serum and testes and dietary Sr intake in normal rats. This is consistent with the results of previous studies. However, we found that there was no linear correlation between sperm quality and serum or testicular Sr in rats. To better explain the problem, we additionally examined the direct effect of different amounts of SrCl_2_ on the cell viability of TM4 cells. This experiment was presented as a supplementary experiment in the Supplementary Materials (Figure S1). It was found that the cell viability of TM4 cells was enhanced with increasing Sr concentration within a certain concentration range. However, when the Sr content was excessive, the cell viability of TM4 cells was suppressed. We therefore hypothesize that SrCl_2_ may affect sperm quality in rats by affecting the function of TM4 cells. Therefore, it is necessary to explore the safe limits of SrCl_2_ for its use in reproduction.

T is the dominant androgen in the testes and is essential for spermatogenesis and male fertility [41]. In this study, we observed dose-dependent increases in serum and testicular T levels as the concentration of SrCl_2_ was increased during feeding. However, sperm quality showed a tendency to increase and then decrease with increasing concentration. Furthermore, we observed that T, but not semen quality, was significantly and positively correlated with Sr levels in both serum and testes. A plausible explanation is that there are multiple pathways affecting spermatogenesis, and we hypothesize that SrCl_2_ may not improve sperm quality by affecting the expression of steroid hormone levels. The exact mechanism remains to be dissected further.

ACP is associated with the degeneration of spermatophores and phagocytosis of Sertoli cells, and its activity can be used to measure the occurrence of spermatogenesis disorders [42]. In this study, supplementing Sr can increase ACP content in the testis of rats, and the effect of the Sr-50 group was significant (*p* < 0.05). It was shown that Sr can play a role in spermatogenesis by facilitating cell division. LDH and SDH are widely distributed in seminiferous tubules and germ cells, which are essential for the energy metabolism of sperm and the growth and development of testicular cells [43]. Previous studies have reported that SDH is primarily found in the mitochondria of Sertoli cells and spermatozoa, and can convert sorbitol into fructose to provide energy for sperm cells [44]. LDH not only provides energy for spermatogenesis by catalyzing glucose metabolism but is also involved in the maturation of spermatogenic cells, testis, and sperm [45]. Previous studies have found that FDP-Sr reduces cyclophosphamide-induced reproductive toxicity in rats by increasing the activity of LDH and SDH [46]. In this study, we found that Sr treatment increased LDH activity in rat testes, with significant effects in the Sr-50 group (*p* < 0.05). Sr treatment could increase the activity of SDH, but there was no significant difference among the three groups (*p* > 0.05). This is consistent with previous studies. NOS is widely distributed in the testis, epididymis, and vas deferens, and is a sensitive index to judge the reproductive toxicity of drugs in male rats. NO can directly or indirectly damage the male reproductive system and affect the spermatogenesis and capacitation process [47]. Our results show that the NOS content does not change significantly after Sr treatment, while the NO content decreases in a dose-dependent manner (*p* < 0.05). The above results showed that Sr exposure may have beneficial effects on male reproductive health in normal hosts.

In addition to regulating host health, gut microbes also play a key role in the relationship between diet and host. Studies have reported that stable gut microbiota not only promoted the absorption of nutrients (such as mineral elements and vitamins) needed for sperm health formation but also prevented toxic and harmful substances such as toxins from crossing the intestinal barrier into the bloodstream, thus maintaining or even increasing their sperm count and vitality [48]. A recent study showed that the balance between Sr and Ca mainly depended on intestinal absorption [49]. Sr and Ca are in a dynamic equilibrium state, and the effect of Sr on the body is affected by the Ca/Sr ratio. When homeostasis is disrupted, a decrease in the ratio may have adverse effects on the body.

To study the effects of Sr on the gut microbiota of a normal host, we conducted 16S rDNA sequencing of the colon content of rats. Surprisingly, the Alpha diversity index results showed that Sr exposure did not affect the richness and diversity of intestinal flora in normal rats. NMDS results showed significant separation between the Sr-200 group and the CTL group, indicating that Sr could affect the intestinal flora structure of normal hosts. Among them, we focused on the analysis of bacteria at the phylum level (top 10) and genus level (top 32), including *Bacteroidetes*, *Tenericutes*, *Romboutsia*, *Ruminococcaceae_UCG-014*, *Weissella*, *Eubacterium_coprostanoligenes_group*, and *Jeotgalicoccus*. Additionally, as the main intestinal flora, *Firmicutes* and *Bacteroidetes* participate in many key functions of the host, such as development, metabolism, and immune function [50]. A recent study found that *Bacteroidetes* was negatively correlated with testicular function, while testicular health was positively associated with the *Firm/Bac* ratio [32]. *Romboutsia* has been reported to be important in maintaining the host’s health [51]. A recent study showed that *Romboutsia* was negatively associated with improved sperm quality [52]. *Ruminococcaceae_UCG-014* has been reported to destroy intestinal homeostasis in mice [53]. Bacteriocin peptides from *Weissella* have been found to affect sperm motility and even exhibit spermicidal activity. The role of *Tenericutes* and *Eubacterium_coprostanoligenes_group* in male reproductive health has not yet been reported. Spearman correlation analysis showed that *Bacteroidetes*, *Tenericutes*, *Romboutsia*, *Ruminococcaceae_UCG-014*, *Weissella*, and *Eubacterium_coprostanoligenes_group* were negatively correlated with sex hormone levels, and significantly positively correlated with indicators of damaging effects on the reproductive system such as NO and NOS (*p* < 0.05), consistent with previous reports. *Tenericutes* and *Eubacterium_coprostanoligenes_group* were found to be negatively correlated with testicular function for the first time. Previous studies have found that *Jeotgalicoccus* reportedly plays a vital role in physical health and was regarded as a potentially beneficial provider of physical services [54]. Zheng et al. [55] found that high concentrations of Cu can cause tissue damage, affect microbial balance, and reduce the relative expression abundance of *Jeotgalicoccu*. In our study, we found a significant positive correlation between *Jeotgalicoccu* and LH (*p* < 0.05). In addition, we also found a positive correlation between *Jeotgalicoccu* and sperm motility, sperm viability, and T levels (*p* > 0.05), but not statistically significant. It is the only bacterium that has shown a positive effect on the reproductive health of normal rats, suggesting that *Jeotgalicoccus* may be important in preventing or treating male reproductive decline. Moreover, we found that most of the bacteria have significant correlations with each other. Therefore, we speculate that after Sr treatment, the bacteria can influence each other, working together to maintain the homeostasis of the gut flora. To our surprise, we found that there was no significant correlation between these bacteria and sperm quality parameters, which may be related to the small sample size of our 16S rDNA sequencing. In the future, we need to further explore the effect of intestinal flora in mediating SrCl_2_ on male reproductive function.

To explore how Sr regulates reproductive health. We detected Sr in serum and testicular tissue, which found that treatment with Sr significantly increased the amount of Sr in serum and testes in a dose-dependent manner. This suggests that Sr can get into the bloodstream through the rat’s intestinal tract, where it can be transported and accumulated to the testes and other reproductive organs. The bacteria most related to phenotype (*Jeotgalicoccus*, *Bacteroidetes*, *Ruminococcaceae_UCG_014*, and *Weissella*) were analyzed by linear regression with Sr in serum and testis. It was found that these bacteria are closely related to Sr in serum and testes. *Jeotgalicoccus* is positively correlated with Sr in serum and testis, while *Bacteroidetes*, *Ruminococcaceae_UCG_014*, and *Weissella* are negatively correlated with Sr in serum and testis. However, the relationship between Sr content (serum and testes) and *Jeotgalicoccus* was opposite to that of the other three. These results suggest that Sr can modulate the relative abundances of *Jeotgalicoccus*, *Bacteroidetes*, *Ruminococcaceae_UCG_014*, and *Weissella*. However, whether Sr can play a role through intestinal flora remains to be further explored. At present, 16S rDNA sequencing is only a relatively quantitative method, and further functional validation is needed in the future to more accurately understand the effect of Sr on the functional activity of the gut microbiome (methods such as fecal transplantation and antibiotics).

## 4. Materials and Methods

### 4.1. Reagents and Chemicals

Analytical grade SrCl_2_·6H_2_O (cat#AKSL-051059) was purchased from Beijing Aikesailun Biotechnology Co., Ltd. (Beijing, China). The enzyme-linked immunosorbent assay (ELISA) kits of T (cat#MM-0577R2), LH (cat#MM-0624R2), FSH (cat#MM-70867R2), ACP (cat#MM-71090R2), LDH (cat#MM-0605R2), SDH (cat#MM-20910R2), NOS (cat#MM-0451R2), and NO (cat#ADS-W-N005-96) were all supplied by Jiangsu Meimian Industrial Co., Ltd. (Jiangsu, China). Animal testicular tissue fixative solution (cat#G1121-500ML) was obtained from Wuhan Servicebio Technology Co., Ltd. (Wuhan, China). Unless otherwise stated, all chemicals used in this study were of analytical grade.

### 4.2. Animals and Experimental Design

**Figure 7:**
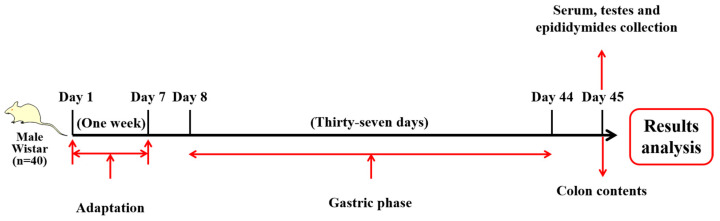
Experimental design.

Forty male specific pathogen-free (SPF) Wistar rats (6–8 weeks, 250 ± 20 g) were obtained from Beijing Vital River Laboratory Animal Technology Co., Ltd. (Beijing, China) and reared under standard laboratory conditions (12 h light/dark cycle, temperature 23 ± 2 °C, humidity 50–60%) with free access to food (chow diet) and water. After 1 week of adaptation, the rats were randomly assigned into four groups (*n* = 10 rats per group): (1) CTL (vehicle control, ddH_2_O); (2) Sr-50 (SrCl_2_·6H_2_O 50 mg/kg BW); (3) Sr-100 (SrCl_2_·6H_2_O 100 mg/kg BW); and (4) Sr-200 (SrCl_2_·6H_2_O 200 mg/kg BW). CTL, Sr-50, Sr-100, and Sr-200 were gavaged once a day (2 mL/rat) for 37 days. Body weight was measured every 3 days. Before dissection, the rats were fasted for 12 h and anesthetized by ether. To minimize suffering, the rats were sacrificed by cervical dislocation and serum, testes, epididymides, and colon contents were collected for further analyses. All experimental procedures and conditions were approved by the Institutional Animal Care and Use Committee at the Institute of Animal Sciences of the Chinese Academy of Agricultural Sciences (IAS2022-145). The experimental design chart is shown below (Figure 7).

### 4.3. Sperm Parameter Assessment and Measurement of Hormone Levels

After the rats were euthanized, both sides of the cauda epididymides were removed, cut into small pieces with a scalpel, and maintained in DMEM/F12 medium with 10% fetal bovine serum (FBS) for 10 min at 37 °C incubator to make a sperm suspension. The number of live and dead sperm was counted, and the sperm quality was calculated by Zhongke Hengye Sperm Automatic Detection and Analysis System ZKPACS-E Type (Beijing Zhongke Hengye Technology Co., Ltd. (Beijing, China)) to calculate sperm concentration, motility, and viability. The hormone levels of T (serum and testis), LH, and FSH were detected with ELISA kits.

### 4.4. Histological Analysis

After dissection, testicular tissues were immediately fixed in a fixative solution, dehydrated in graded ethanol, cleared through xylene, paraffin-embedded, and sectioned (3 μm thick). Testicular histopathology was analyzed by hematoxylin and eosin staining (H&E).

### 4.5. Biochemical Indicator Analysis

An appropriate amount of testicular tissue was added to phosphate-buffered saline (PBS) (0.01 mol/L) at a ratio of 1:9 (m/V). The testicular tissue was ground in an ice bath with a tissue homogenizer and centrifuged at 5000 rpm for 15 min. The supernatant was obtained for further measurement. The level of NO in testicular tissue was measured by nitrate reductase method, and the contents of ACP, LDH, SDH, and NOS levels in the testicular tissue of rats were detected by ELISA according to the manufacturer’s instructions.

### 4.6. Detection of Sr Content

Minor and trace element content Sr was determined using a Quadrupole ICP-MS (Agilent 7700×, Agilent Technologies Inc., Santa Clara, CA, USA) instrument. Serum (100 μL) and testis (100 mg) were added to a microwave digestion tube, followed by the addition of 5 mL of 65% nitric acid and 3 mL of 30% hydrogen peroxide. The mixture was left overnight at room temperature before being covered with shrapnel and tightened with an outer cover. Subsequently, the sample was subjected to controlled digestion at a temperature of 190 °C in a microwave digester after a pre-digestion blank test. Upon completion, the resulting solution was transferred into a constant volume of 50 mL, mixed thoroughly, and set aside.

### 4.7. Gene Expression Analysis by RT-qPCR

Total RNA was isolated from testicular tissue (100 mg) using TRIzol reagent (Thermo Fisher Scientific, Waltham, MA, USA). After total RNA extraction, reverse transcription was performed using PrimeScript™ RT (Takara, Dalian, China) reagent Kit to obtain cDNA. TB Green Premix Ex Taq II (Takara, Dalian, China) was used to detect gene expression of a marker of testosterone biosynthesis (*STAR*, *3β-HSD*, *Cyp11a1*, and *Cyp17a1*) with the Bio-Rad CFX96 system (Bio-Rad, Hercules, CA, USA). Using glyceraldehyde phosphate dehydrogenase (*GAPDH*) as the housekeeping gene, the relative expression levels of target genes were calculated using the 2^−ΔΔCt^ method. Primer sequences used for RT-qPCR are shown in Table 1.

### 4.8. Intestinal Microbial DNA Extraction and 16S rDNA Sequencing

HiPure Soil DNA Extraction kit (Magen, Guangzhou, China) was used to extract microbial DNA from colon contents (*n* = 5/group). The 16S rRNA sequencing genes (V3–V4) were amplified using the 341F/806R primers pair (341F: 5′-CCTACGGGNGGCWGCAG-3′; 806R: 5′-GGACTACHVGGGTATCTAAT-3′) in triplicate. Amplification products were assessed for quality using 2% agarose gel electrophoresis and AxyPrep DNA Gel Extraction Kit (Axygen Biosciences, Union City, CA, USA) and were purified according to the manufacturer’s instructions. Quantification was performed using the ABI StepOnePlus Real-Time PCR System (Life Technologies, Foster City, CA, USA).

### 4.9. TM4 Cell Culture and Treatment

Mouse TM4 cells were purchased from Jiangsu Aidisheng Biological Technology Co., Ltd. (Jiangsu, China). TM4 cells were cultured in DMEM medium containing 10% FBS at 37 °C and 5% CO_2_. TM4 cells at a concentration of 1 × 10^5^ cells/well in 96-well plates. After the cells were attached, the cells were treated with different concentrations of SrCl_2_·6H_2_O (0, 0.01, 0.05, 0.1, 0.5, 1, 5, 10, 20, 30, 40 mM) for 24 h, and the cell viability was evaluated by Cell Counting Kit-8 (CCK-8).

### 4.10. Statistical Analysis

The data are expressed as the mean ± standard error of the mean (SEM) of at least three independent experiments. Except for gut microbiota sequencing, data were analyzed using GraphPad Prism 8.0 (GraphPad Software, San Diego, CA, USA). One-way ANOVA test and Tukey’s multiple comparison tests were used to evaluate whether there was statistical significance between groups. The gut microbiota sequences were examined by rank sum test, while the two groups were compared by the Wilcoxon rank sum test. When the group size reached three or more, the Kruskal–Wallis rank sum test was used. Correlations were statistically evaluated using Spearman’s correlation tests with Origin Pro 2022 (OriginLab Corporation, Northampton, MA, USA). *p* < 0.05 was considered a statistically significant difference between the control and Sr-exposed groups.

## 5. Conclusions

**Figure 8:**
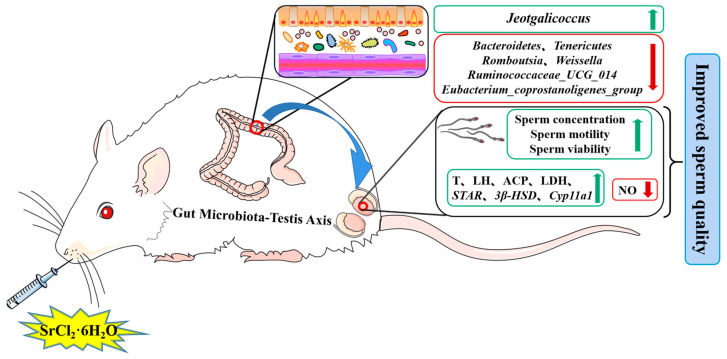
Mechanism of SrCl_2_ effects on male reproductive function and gut microbiota. The up (↑) and down (↓) arrows indicate increased or decreased of its expression, respectively.

In conclusion, according to the phenotypic results of Wistar rats in this experiment, we found that Sr treatment had no effect on the weight of normal rats. The Sr-50 group significantly increased T levels and sperm quality, and improved the levels of testicular marker enzymes and testosterone marker genes in the rats. Compared with the CTL group, there was no significant difference in sperm quality parameters between the Sr-100 group and the Sr-200 group. The above results suggest that Sr has great potential for maintaining and improving male reproductive function. In addition, the underlying mechanism was explored by 16S rDNA (Figure 8). It was found that *Bacteroidetes* and *Tenericutes*, *Romboutsia*, *Ruminococcaceae_UCG_014*, *Weissella*, *Eubacterium_coprostanoligenes_group*, and *Jeotgalicoccu* are potentially key bacteria associated with the reproductive health of normal male rats. However, its specific mechanism needs to be further studied and verified. At present, there is still no clear regulation of the Sr limit. Our study provides a reference for the safety of SrCl_2_, and provides evidence for the development of Sr-rich foods and their prevention of male fertility decline.

## Figures and Tables

**Table 1:** The primer sequence for Real-Time qPCR.

Target Gene	Primer Direction	Sequences (5′ to 3′)
*STAR*	Forward	CCCAAATGTCAAGGAAATCA
	Reverse	AGGCATCTCCCCAAAGTG
*3β-HSD*	Forward	CCCTGCTCTACTGGCTTGC
	Reverse	TCTGCTTGGCTTCCTCCC
*Cyp11a1*	Forward	AAGTATCCGTGATGTGGG
	Reverse	TCATACAGTGTCGCCTTTTCT
*Cyp17a1*	Forward	TGGCTTTCCTGGTGCACAATC
	Reverse	TGAAAGTTGGTGTTCGGCTGAAG
*GAPDH*	Forward	TCAAGAAGGTGGTGAAGCAG
	Reverse	AAGGTGGAAGAGTGGGAGTTG

## Data Availability

All data generated or analyzed during this study are included in this article, and materials are available from the authors upon reasonable request.

## Supplementary Materials

The following supporting information can be downloaded at https://www.mdpi.com/article/10.3390/ijms241813922/s1.
