# Supplementary material for: Strontium Chloride Improves Reproductive Function and Alters Gut Microbiota in Male Rats
Source: Int J Mol Sci. 2023 Sep 10;24(18):13922. doi: 10.3390/ijms241813922 (PMC10531462; doi:10.3390/ijms241813922)
Supplement: Supplementary file 1 [file ijms-24-13922-s001.zip › ijms-2570790-supplementary.pdf]

Supplementary Material

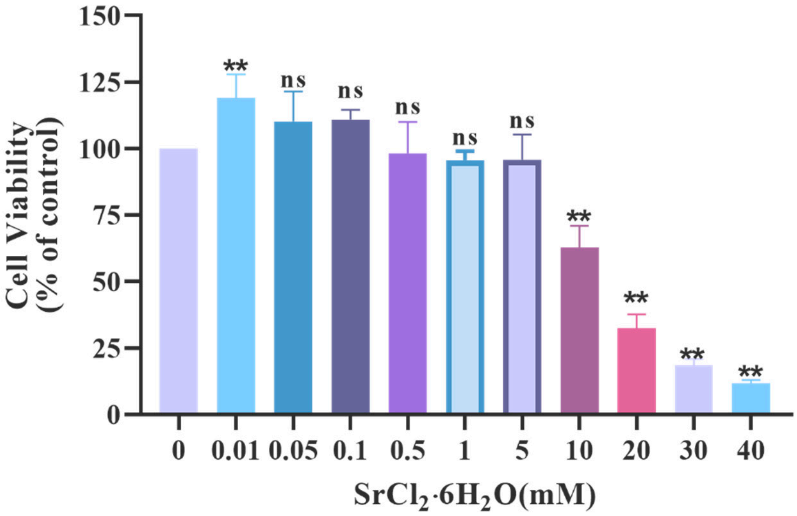

Figure S1. Effect of $SrCl_2·6H_2O$ on cell viability of TM4 cells. Cell viability was detected using CCK-8 assay. Compared with the control group, * $p < 0.05$ and ** $p < 0.01$.
